# Editorial: Extracellular Vesicles as Next Generation Therapeutics

**DOI:** 10.3389/fcell.2022.919426

**Published:** 2022-05-12

**Authors:** Sylwia Bobis-Wozowicz, Eduardo Marbán

**Affiliations:** ^1^ Department of Cell Biology, Faculty of Biochemistry, Biophysics and Biotechnology, Jagiellonian University, Krakow, Poland; ^2^ Smidt Heart Institute, Cedars Sinai Medical Center, Los Angeles, CA, United States

**Keywords:** extracellular vesicles, therapy, regeneration, disease treatment, drug delivery, vaccine, bioengineering, nanomedicine

Extracellular vesicles (EVs) are nano-sized (30–200 nm) cell membrane-derived circular structures, released by virtually any cell type. They carry variety of bioactive molecules, including proteins, lipids and RNA species, by which they can influence phenotype and properties of other cells. Owing to such properties, EVs are increasingly recognized as important mediators of cell-to-cell communication system, which can be successfully leveraged for therapeutical use in the treatment of human diseases (Wiklander et al., 2019). This Research Topic aimed at presenting recent advances in utilizing EVs as future medicines, regarding different aspects of EVs engineering, manufacturing and testing their therapeutic efficacy.

**FIGURE 1:**
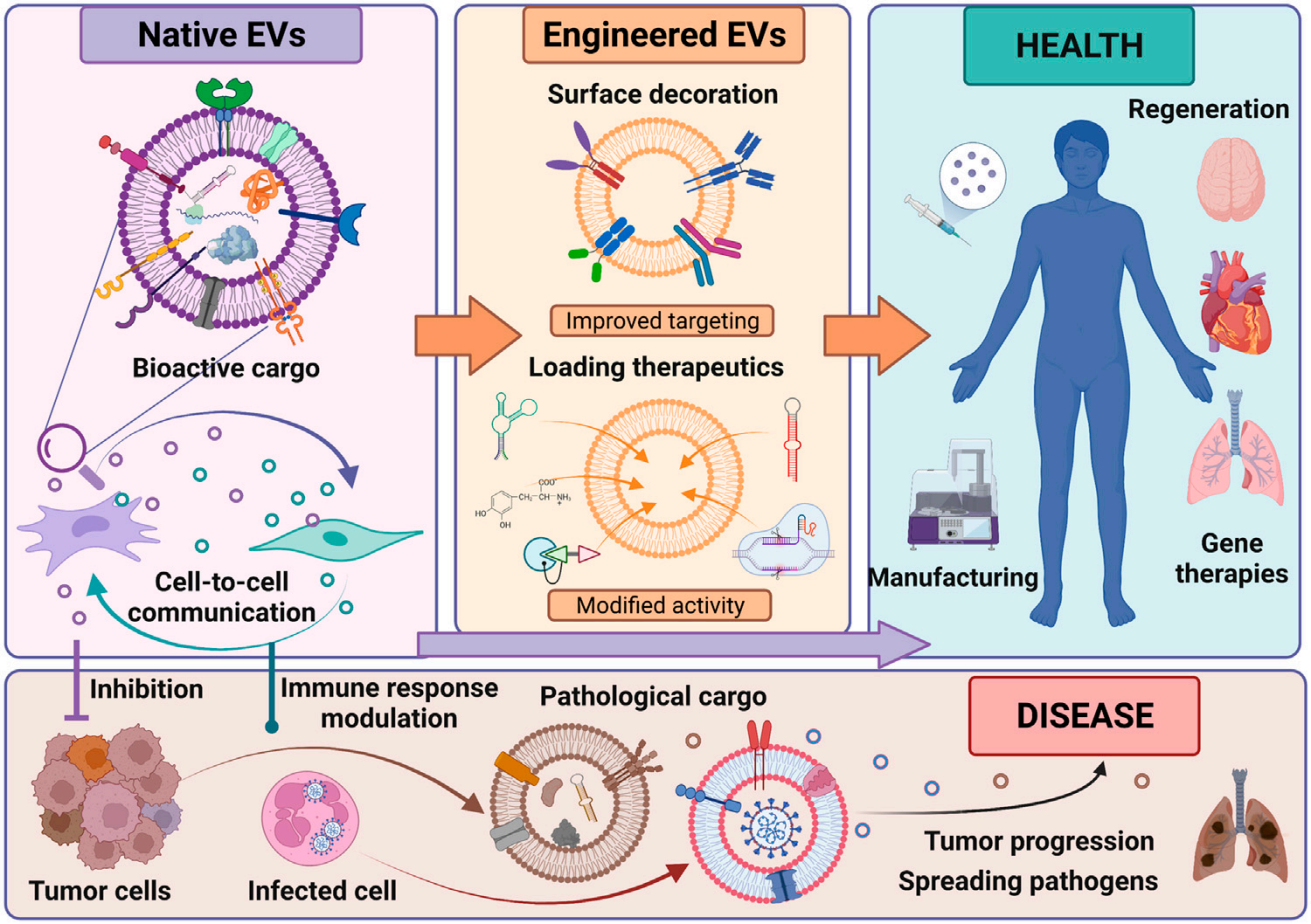
Characteristics, bioengineering and role of extracellular vesicles (EVs) in health and disease. EVs contain bioactive cargo in the form of proteins, lipids and nucleic acids which exert biological effects on target cells and thereby influence their fate. To improve functionality and specificity of EVs, they can be engineered by surface modification and/or loading a specific therapeutic cargo. Native and modified EVs can be used as next-generation cell-free therapeutics to improve health. On the contrary, EVs released in pathological conditions by, e.g. cancer cells or infected cells, participate in disease spreading and progression.

We have collected four original articles, four reviews and two perspectives, which cover the most important aspects of biomedical applications of EVs and EV-derived bioactive molecules (Figure 1). The list of medical indications, which can benefit from EVs usage, is increasingly growing. Some of these areas are considered in this article collection, including heart disease, stroke, pulmonary fibrosis, dermatological conditions, immunomodulation and cancer. However, the information provided here does not fully exhaust the broad possibilities of EV utility as next-generation therapeutics.

A comprehensive review on the recent progress in clinical applications of EVs, bioengineering and the complexity of EV-molecular cargo, is presented by Claridge et al. The authors mark new avenues for future work related to EVs, with the goal to improve EV-based therapeutic strategies. Importantly, current limitations of EV utility are also discussed, such as manufacturing, scalability, molecular characterization and targeted delivery.

Further elaboration on therapeutic potential of exosomes is provided in the review by Shi et al. The authors focused on dermatological conditions, including wound recovery, burning, senescence and scars. They comment on regulatory function of exosomes in skin development and implicate a novel role of exosomes as biomarkers of cutaneous diseases. In turn, Wan et al. discuss the hurdles and perspectives for EV therapy in the treatment of stroke. They outline an important role of microglia in neurogenesis and neuroinflammation, including pathogenesis of stroke. Importantly, EVs from different sources may affect the activity of microglia and ameliorate stroke progression.

Apart from native cargo, bioactive content of EVs can also be modified to enhance their pro-regenerative capacity. This approach was taken by Ibrahim et al. On the basis of their prior work, the authors introduced beta-catenin and gata4 into therapeutically inert fibroblasts, to obtain activated specialized tissue effector EVs (ASTEX) with proven pro-regenerative capacity. ASTEX were able to reduce fibrotic markers in fibroblasts and endothelial cells, *via* the activity of anti-fibrotic miRNAs. Application of ASTEX in bleomycin induced lung damage, exerted beneficial effects in the experimental animals, both short- and long-term, proving their utility as anti-fibrotic agents.

Similarly, pro-regenerative activity another miRNA molecule contained in the EVs, was investigated by Sanchez-Sanchez et. al. They explored cardioprotective properties of hypoxia-stimulated mesenchymal stem cell (MSC)-derived EV-containing miR-4732-3p. Activity of this miRNA was demonstrated to promote angiogenesis and ameliorate fibrosis, both *in vitro* and *in vivo,* in a rat model of myocardial infarction.

EVs were shown not only to directly stimulate tissue-residing cells for regeneration, but also to modulate function of immune cells, which are the major players in tissue injury and resolution. With this respect, Peck et al. showed that introducing a specific molecule exerting immunomodulatory function in neutral fibroblasts, resulted in the production of EVs with enhanced immunomodulatory capacity. Specifically, they selected tryptophan 2,3-dioxygenase (TDO2), a target gene of canonical Wnt-β-catenin pathway. Applying TDO2-enriched EVs in an animal model of acute myocardial infarction, greatly improved heart function.

Another dimension of immunomodulatory activity of EVs was presented by Cochran and Kornbluth, in the context of fighting cancer. EVs isolated from immune cells—natural killer NK3.3 cell line, were shown to contain an array of cytotoxic enzymes, as well as variety of miRNAs associated with anti-tumor activity. In a cell killing assay, such EVs were able to inhibit proliferation and induce apoptosis in hematopoietic and non-hematopoietic tumor cell lines. Importantly, the observed effect was tumor cell specific, since normal cells remained unaffected.

With respect to tumor cells, however, it was shown that they produce EVs which promote tumor cell proliferation and metastasis, leading to tumor progression (Figure 1). Such EVs can alter tumor microenvironment, allowing tumor cells to escape recognition and clearance by immune cells (Xu et al., 2018). A summary of recent findings in the field of tumor-related EVs, with emphasis on oral squamous cell carcinoma (OSCC), is presented in a review by Lu et al. OSCC represents 90% of oral cancers and is associated with low survival rate, thus constitutes a serious health problem. One of novel treatment options for OSCC may constitute EV isolated from therapeutically-approved cells, such as MSCs.

Apart from tumor cells, pathological EVs can also be released during inflammation and they are involved in spreading pathogens. In the light of recent SARS-CoV-2 pandemic, which affected millions of individuals worldwide and resulted in the onset of COVID-19 disease, the contribution by Pironti et al., provides a timely discussion on the potential link between virus spreading *via* EV secretory pathway and cardiovascular manifestations of COVID-19. It has been investigated that COVID-19 disturbs the renin-angiotensin system, thereby exacerbating heart function. To improve heart condition, EVs derived from therapeutically beneficial cells, such as MSCs or cardiosphere-derived cells (CDCs), can be applied.

The Research Topic of EV engineering, to enhance their biological activity and improve targeting toward specific cell or tissue type is comprehensively discussed by Nazimek and Bryniarski. It is known that upon delivery to a living organism, EVs are rapidly cleared by phagocytes, which significantly reduces their therapeutic potential. As presented by Nazimek et al., EVs co-incubation with antigen-specific antibodies and light chains induces their aggregation. This modification extends their stability in body fluids and allows their escape from urinary excretion. Moreover, EV decoration with a specific antibody can greatly enhance tissue targeting. These advancements may boost therapeutic potential of EVs and their applicability to treat human diseases.

Concluding, this Research Topic provides the state-of-the-art of recent discoveries in the field of EVs and highlights the importance of further research needed to fully explore their therapeutic potential. Transferring this knowledge to clinical practice will be a milestone achievement in contemporary medicine.
